# Renal cancer secretome induces migration of mesenchymal stromal cells

**DOI:** 10.1186/s13287-023-03430-4

**Published:** 2023-08-10

**Authors:** Piotr Popławski, Weronika Zarychta-Wiśniewska, Anna Burdzińska, Joanna Bogusławska, Anna Adamiok-Ostrowska, Karolina Hanusek, Beata Rybicka, Alex Białas, Helena Kossowska, Roksana Iwanicka-Nowicka, Marta Koblowska, Leszek Pączek, Agnieszka Piekiełko-Witkowska

**Affiliations:** 1 Department of Biochemistry and Molecular Biology, Centre of Postgraduate Medical Education, Warsaw, Poland; 2 Present Address: Department of Immunology, Transplantology and Internal Diseases, Medical University of Warsaw, Warsaw, Poland; 3 Department of Physiological Sciences, Institute of Veterinary Medicine, Warsaw University of Life Sciences, Warsaw, Poland; 4 Laboratory of Systems Biology, Faculty of Biology, University of Warsaw, 02-106 Warsaw, Poland; 5 Laboratory of Microarray Analysis, Institute of Biochemistry and Biophysics, Polish Academy of Sciences, Warsaw, Poland; 6 Institute of Biochemistry and Biophysics, Polish Academy of Sciences, Warsaw, Poland

**Keywords:** Renal cell cancer, Mesenchymal stromal cells, AREG, DPP4, FN1

## Abstract

**Background:**

Advanced renal cell carcinoma (RCC) is therapeutically challenging. RCC progression is facilitated by mesenchymal stem/stromal cells (MSCs) that exert remarkable tumor tropism. The specific mechanisms mediating MSCs’ migration to RCC remain unknown. Here, we aimed to comprehensively analyze RCC secretome to identify MSCs attractants.

**Methods:**

Conditioned media (CM) were collected from five RCC-derived cell lines (Caki-1, 786-O, A498, KIJ265T and KIJ308T) and non-tumorous control cell line (RPTEC/TERT1) and analyzed using cytokine arrays targeting 274 cytokines in addition to global CM proteomics. MSCs were isolated from bone marrow of patients undergoing standard orthopedic surgeries. RCC CM and the selected recombinant cytokines were used to analyze their influence on MSCs migration and microarray-targeted gene expression. The expression of genes encoding cytokines was evaluated in 100 matched-paired control-RCC tumor samples.

**Results:**

When compared with normal cells, CM from advanced RCC cell lines (Caki-1 and KIJ265T) were the strongest stimulators of MSCs migration. Targeted analysis of 274 cytokines and global proteomics of RCC CM revealed decreased DPP4 and EGF, as well as increased AREG, FN1 and MMP1, with consistently altered gene expression in RCC cell lines and tumors. AREG and FN1 stimulated, while DPP4 attenuated MSCs migration. RCC CM induced MSCs’ transcriptional reprogramming, stimulating the expression of CD44, PTX3 and RAB27B. RCC cells secreted hyaluronic acid (HA), a CD44 ligand mediating MSCs’ homing to the kidney. AREG emerged as an upregulator of MSCs’ transcription.

**Conclusions:**

Advanced RCC cells secrete AREG, FN1 and HA to induce MSCs migration, while DPP4 loss prevents its inhibitory effect on MSCs homing. RCC secretome induces MSCs’ transcriptional reprograming to facilitate their migration. The identified components of RCC secretome represent potential therapeutic targets.

**Graphical abstract:**

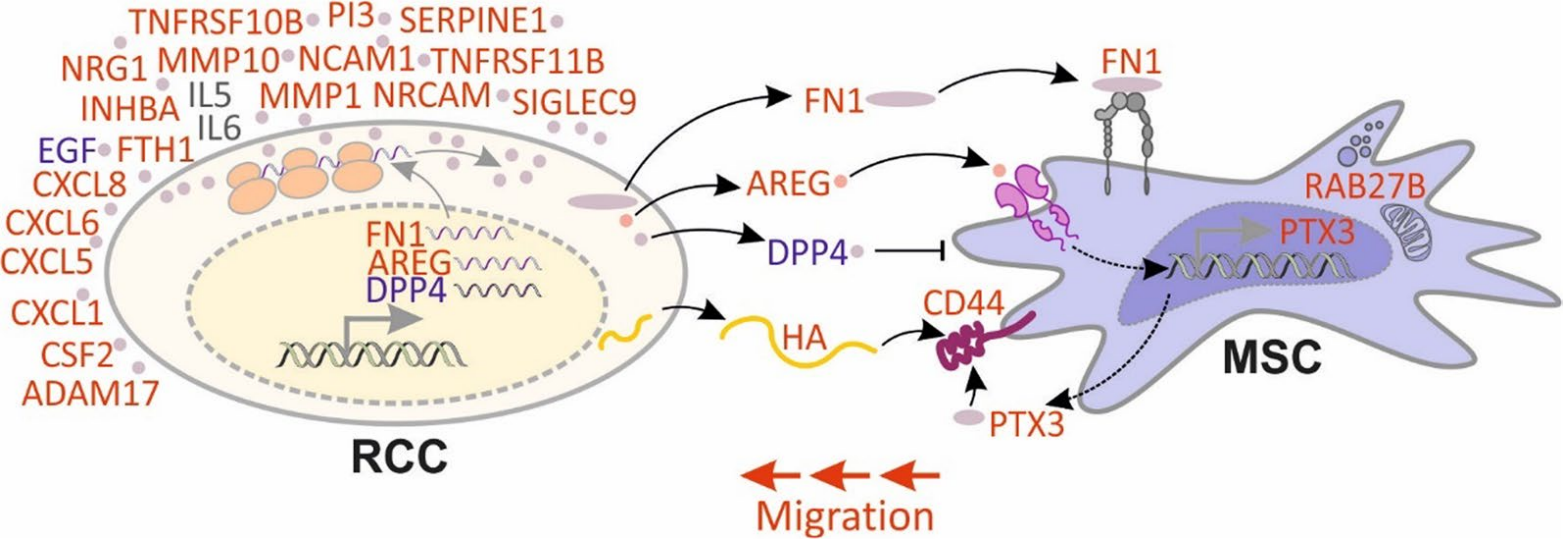

**Supplementary Information:**

The online version contains supplementary material available at 10.1186/s13287-023-03430-4.

## Highlights

- Mesenchymal stem/stromal cells (MSCs) facilitate renal cell cancer (RCC) progression
- RCC secretes high amounts of AREG and FN1
- DPP4 secretion is attenuated in RCC cells
- AREG and FN1 stimulate, while DPP4 attenuates MSCs migration
- RCC secretome reprograms MSCs transcription to facilitate their migration

**Supplementary Information:**

The online version contains supplementary material available at 10.1186/s13287-023-03430-4.

## Background

Renal cell carcinoma (RCC) affects 400,000 people annually worldwide, making it the most common malignancy of the kidney [1]. The prognosis for early-stage RCC is good, with more than 90% of patients surviving within 5 years. Unfortunately, metastatic RCC (mRCC), which occurs in about one-third of the patients, is still therapeutically challenging. Despite growing treatment options (anti-angiogenic therapy and immune checkpoint inhibitors), most patients with mRCC inevitably relapse with progression, resulting in less than 30% 5-year survival [1].

Multipotent mesenchymal stem/stromal cells (MSCs) can differentiate into several cell lineages, including osteoblasts, chondrocytes and adipocytes, and play an important role in various processes, including wound healing, inflammation or angiogenesis [2, 3]. MSCs exert remarkable tropism to tumors, induced by various types of chemokines, cytokines, inflammatory factors or growth factors [4]. MSCs’ ability to accumulate at tumor sites is utilized for the selective, tumor-specific delivery of anticancer drugs. Such new ways of drug delivery are currently tested in clinical trials [5]. On the other hand, MSCs themselves can actively influence tumor progression. Depending on the study and tumor type, MSCs were reported to promote cancer progression (e.g., by stimulating angiogenesis, immunosuppression or epithelial-mesenchymal transition) or to attenuate tumor growth by suppressing the aforementioned processes [4]. Therefore, delineation of the molecular mechanisms that govern recruitment of MSCs to tumor tissues is crucial for the development of new cancer treatment methods.

Several studies showed that MSCs promote RCC progression. MSCs stimulate proliferation and migration of RCC cells in vitro and facilitate RCC tumor formation in vivo [6, 7]. The studies on the recruitment of MSCs to RCC tumors are scarce and limited mainly to MSCs engineered to carry anticancer drugs. Such modified MSCs attenuated renal cancer progression in mice models and were selectively recruited to RCC tumors and/or metastatic sites in mice [8, 9]. However, the specific mechanisms mediating MSCs tropism to RCC cells remain largely unknown. Here, we hypothesized that the molecules secreted by RCC cells can affect the functioning of MSCs, regulating their migration and recruitment to RCC tumors. To verify this hypothesis, we aimed to comprehensively analyze the RCC secretome and cytokinome to identify proteins that act as MSCs attractants. Our study shows that RCC cells secrete multiple cytokines and other proteins that affect gene expression and the functioning of MSCs. In particular, we found that RCC cells derived from the RCC tumors consistently secrete high amounts of FN1 and AREG that induce MSCs’ migration. Moreover, the secretion of DPP4, an inhibitor of MSCs’ motility, is diminished in conditioned media from the advanced RCC cell lines. Finally, we found that RCC secretome apparently reprograms MSCs’ gene expression, to facilitate their migration toward cancer cells.

## Material and methods

### Propagation of RCC cell lines and CM collection

RPTEC/TERT1 (CRL-4031, ATCC), Caki-1 (HTB-46, ATCC), 786-O (CRL-1932, ATCC) and A498 (HTB-44, ATCC) were cultured in accordance with manufacturer’s protocol. KIJ265T and KIJ308T cell lines (Mayo Foundation of Medical Education and Research) were cultured as previously described [10]. For the collection of conditioned media (CM), 10^6 cells were seeded at 75cm2 flasks, cultured for 24h and rinsed once with PBS and four times with DMEM (low glucose, no glutamine, no phenol red) (Gibco/Thermo Fisher Scientific, Paisley, UK) with GLUTAMAX (Gibco/Thermo Fisher Scientific, Paisley, UK). Following the addition of 15ml of DMEM (no phenol red) supplemented with GLUTAMAX, the cells were cultured for another 24h. CM were collected, centrifuged, aliquoted and stored at −80 °C. As earlier described [11] CM used for proteomic analysis was filtered by Milex GV Low Protein Binding Durapore (PVDF) 0.22 µm (EMD Millipore Corporation. Billerica, MA). For other experiments, CM was not filtered. Before use in any experiment, CM was centrifuged 10,000 × g for 5 min.

## MSCs’ isolation and propagation

BM-MSCs (bone marrow MSCs) were isolated during standard orthopedic surgeries with the agreement of the Local Bioethics Committee (Approval no. KB/115/2016) and written informed consent of patients (*n* = 7). A bone marrow aspirate was collected to a probe with 500 U of heparin. The cells were cultured in Dulbecco’s modified Eagle’s medium with low glucose (Biowest, Riverside, MO, USA) supplemented with 10% FBS (Biowest, Riverside, MO, USA), Glutamine-Penicillin–Streptomycin (Biowest, Riverside, MO, USA) and amphotericin B (Biowest, Riverside, MO, USA). BM-MSCs’ identity was confirmed using flow-cytometry analysis of surface antigens and differentiation analysis following induction of adipogenesis, chondrogenesis and osteogenesis as previously described [12].

## MSC treatment for microarray and qPCR analyses

50,000 MSCs were seeded on a well of a 12-well plate, cultured for 24h, rinsed twice with DMEM supplemented with GLUTAMAX and cultured for 24h in medium without FBS. Next, the cells were rinsed once with PBS and four times with DMEM supplemented with GLUTAMAX and cultured for 24h in CM from RPTEC/TERT1, Caki-1 or KIJ265T cells.

### Analysis of MSCs’ migration

The influence of RCC CM on MSCs migration was analyzed using Cultrex Cell Migration Assay (Trevigen, Inc., Gaithersburg, MD) following the manufacturer’s protocol. MSCs derived from six patients were cultured to 75% confluence. Next, the culturing was continued for 24h in low-glucose DMEM without phenol red (Thermo Fisher Scientific, Rockford, IL), followed by trypsinization. A total of 20,000 of MSCs in DMEM were added to the upper chamber, with CM used as an attractant. After 24h, the migrated cells were dissociated from the bottom surface of a membrane, incubated with Calcein and measured by fluorescence (485/520 nm). The effect of amphiregulin (AREG), fibronectin (FN1) and matrix metallopeptidase 1 (MMP1) was analyzed using CytoSelect™ 96-Well Cell Migration Assay, 8 µm (Cell Biolabs, Inc., San Diego, CA), following the manufacturer’s protocol. DMEM without phenol red supplemented with Amphiregulin, Fibronectin or MMP1 (all from Sigma-Aldrich, St. Louis, MO) was used for migration analysis. To analyze DPP4 effects, 50,000 of Caki-1 or KIJ265T cells were seeded on 12-well plate well and cultured for 24h, and then DMEM was supplemented with DPP4 (USA R&D Systems, Inc., Minneapolis, MN) and used for analysis of MSC migration. To analyze the effect of MMP1 silencing on MSC, 50,000 of Caki-1 and KIJ265T cells were seeded on 12-well plate, after 24h cells were transfected with silencer select MMP1 siRNA (ID:104016) or control siRNA (Thermo Fisher Scientific, Rockford, IL) using Lipofectamine 2000 (Thermo Fisher Scientific, Rockford, IL). After 24h medium was renewed, and after the additional 24h cells were washed and CM was collected for analysis. MPP1 silencing was verified by qPCR and ELISA.

### Cytokine analysis

A total of 274 cytokines were analyzed in CM using Human Cytokine Array C4000 (RayBiotech, Inc, Norcross, GA). Validation was done using ELISA (Additional file 1: Table S1). DPP4 activity in CM was measured using KA3737 DPP4 Activity Assay Kit (Abnova, Taipei, Taiwan). All tests were done according to the manufacturers’ protocols.

### Isolation of RCC RNA

RNA isolated from RCC tumors and matched-paired non-tumorous control samples was retrieved from the local Department of Biochemistry and Molecular Biology Bank of RNA under approval of the Local Bioethical Committee of Centre of Postgraduate Medical Education with written informed consent of patients (Approval no. 119/PB/2019).

RNA from RCC and normal kidney cell lines was isolated using GeneMATRIX Universal RNA/miRNA Purification Kit (EURX, Gdansk, Poland) following manufacturer’s protocol. RNA concentration was measured with Nanodrop ND-1000 and stored at −80 °C.

### Reverse transcription and qPCR

Reverse transcription and qPCR on RNA isolated from cell lines were performed as previously reported [13]. For tissues, qPCR was performed using TaqMan™ UNIVERSAL Master MIX II (Thermo Fisher Scientific, Rockford, IL). The primers and probes used in the study are provided in Additional file 2: Table S2. Gene expression was normalized to RNA18SN1 and HPRT as described [13].

### Proteomic data

We took advantage of our recently published data on the proteomes of conditioned media from RCC cells [11] (MassIVE repository; dataset identifier PXD030085).

### Microarray analysis

Microarrays were analyzed as previously described [14]. DEGs analysis was performed using IPA software (Qiagen).

### Statistical analysis

All experiments were performed in at least three independent biological repeats. Statistical analysis was performed using ANOVA followed by Dunnett's multiple comparison test, paired t test or Wilcoxon matched pairs test. *p* < 0.05 was considered statistically significant.

## Results

### RCC cell lines isolated from advanced tumors stimulate migration of MSCs

**Fig. 1:**
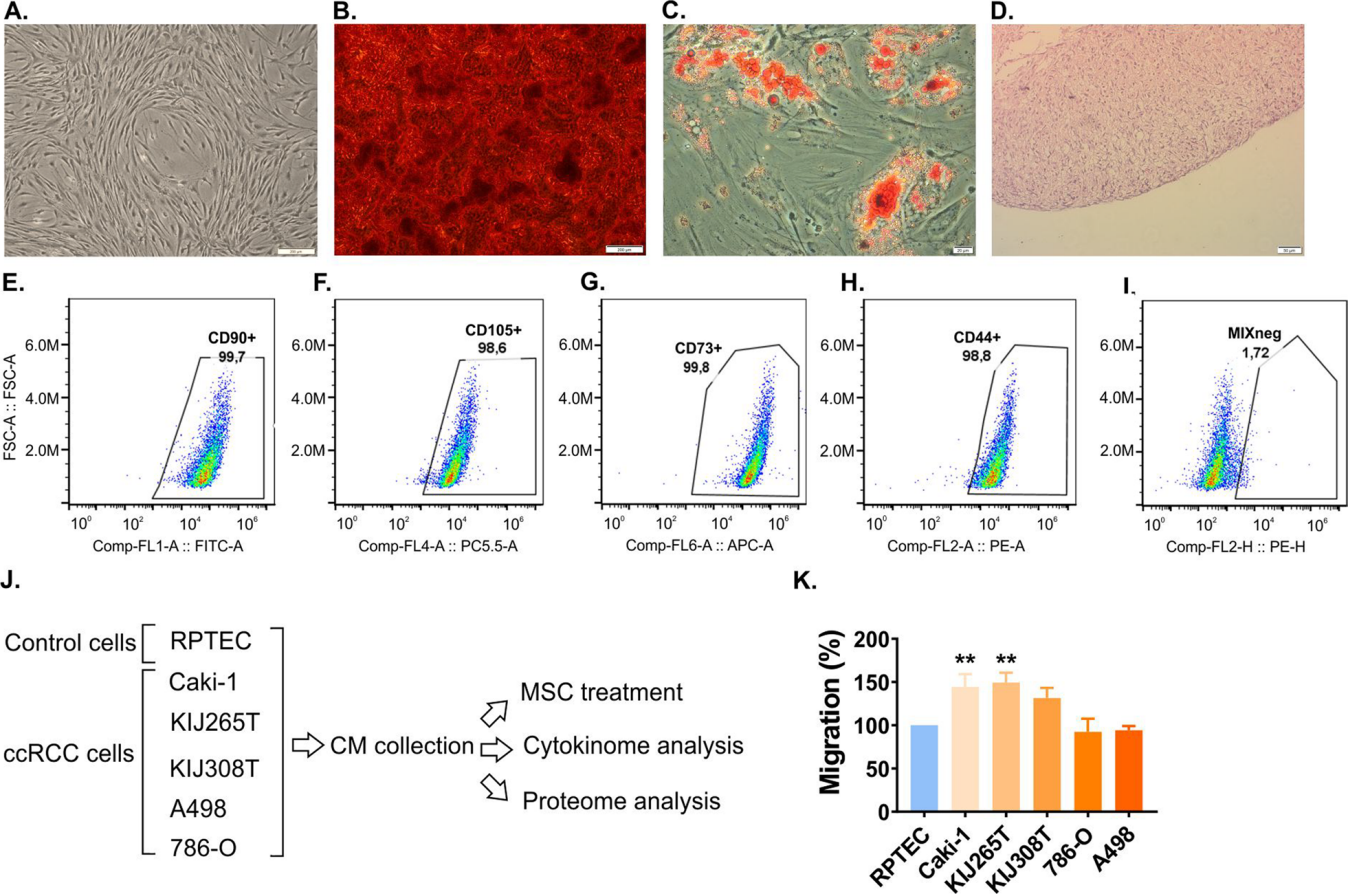
The secretome of the advanced RCC induces MSCs migration. **A** Morphology of undifferentiated MSCs. **B** MSCs osteogenic differentiation: Alizarin Red staining (calcium deposits are red). **C** MSCs adipogenic differentiation: Oil Red O staining (lipid droplets are red). **D** Chondrogenic differentiation: HE staining of chondropellet. Scale bars: 200 μm (A, B), 20 μm (C), 50 μm (D). **E**–**I** The expression of surface antigens on MSCs: CD90 (E), CD105 (F), CD73 (G), CD44 (H) and MIX negative (I) consisting of CD34, CD45, CD11b, CD19 and HLA-DR. **J** The influence of RCC secretome on MSCs: the scheme of the experiment. Conditioned media from five RCC cell lines and normal kidney cell line RPTEC were collected for the tests on the effects on MSCs migration and the analysis of cytokines and proteins. **K** The influence of CM from five RCC cell lines on migration MSCs. The plot shows the results of three independent biological experiments performed on independently treated MSCs derived from six patients. Statistical analysis was performed using ANOVA followed by Dunnett's multiple comparison test. **p < 0.01

Flow-cytometry confirmed that  > 95% of the isolated MSCs did not express hematopoietic markers, while being positive for CD90, CD44, CD105 and CD73 (Fig. 1). The induction into multilineage differentiation revealed the ability of MSCs to differentiate into adipocytes, chondrocytes and osteocytes. To analyze the influence of RCC secretome on MSCs migration, we tested the effects of CM derived from five RCC cell lines. Among all tested cell lines, CM collected from Caki-1 and KIJ265T (RCC skin metastasis and stage IV tumor, respectively) were the strongest stimulators of MSCs migration when compared to non-tumorous control kidney cell lines (Fig. 1).

### RCC cytokinome is altered when compared with normal kidney cells

**Fig. 2:**
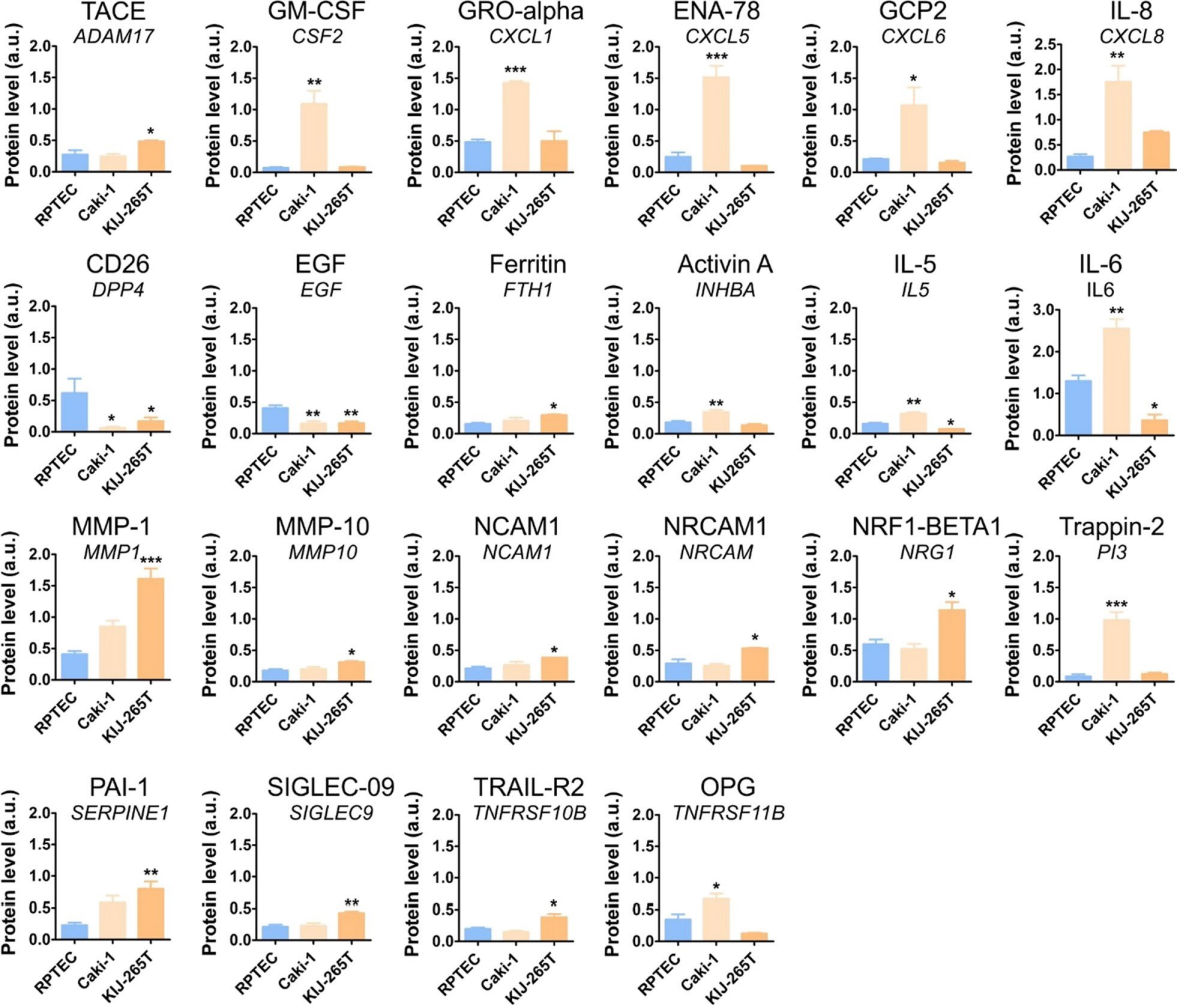
The results of cytokine arrays performed on conditioned media from RCC cells and normal proximal tubules. The plots show the results of densitometric scanning of arrays. N = 3 independent biological experiments. Statistical analysis was performed using ANOVA with Dunnett’s multiple comparison test and t test. *p < 0.05, **p < 0.01, ***p < 0.001. Representative array scans are shown in Additional file 7: Fig. S1

To search for the cytokines that could contribute to the recruitment of MSCs, we performed cytokine arrays targeting the levels of 274 cytokines in CM isolated from Caki-1 and KIJ265T cells, as well as the normal kidney cell line (RPTEC) (Additional file 7: Fig. S1, Additional file 3: Table S3). This revealed statistically significant altered level of 12 and 14 cytokines in CM from Caki-1 cell line and KIJ265T, respectively (Fig. 2). Top altered cytokines in Caki-1 CM included CSF2 (+ 14.7-fold), PI3 (+ 11.73-fold), and DPP4 (-9.75-fold). Top altered cytokines in KIJ265T CM included MMP1 (+ 3.91-fold), SERPINE1 (+ 3.48-fold), and DPP4 (-3.66-fold). Four cytokines (DPP4, EGF, IL5 and IL6) were altered in CM from both analyzed cell lines, with DPP4 and EGF commonly decreased in Caki-1 CM and KIJ265T CM. IL5 and IL6 levels were increased in Caki-1 CM while being decreased in KIJ265T CM (Fig. 2). CXCL8 and MMP1, while being statistically significantly increased in CM from only one RCC cell line, showed a clearly visible upregulation trend in the other analyzed RCC cell line (Fig. 2).

### Advanced RCC secretomes share commonly altered proteins

To extend our search for the potential MSCs attractants, we took advantage of our recent proteomic analysis of CM from different RCC cell lines [11]. AREG and HEBP1 were detected in CM from Caki-1 and KIJ265T cells while being undetectable in CM from RPTEC. FN1 concentration was substantially increased in CM from Caki-1 and KIJ265T cells when compared with RPTEC. Furthermore, in accordance with the results of cytokine arrays, mass spectrometry analysis confirmed the decreased concentrations of DPP4 and increased levels of MMP1 in CM from both cell lines (Additional file 4: Table S4).

**Fig. 3:**
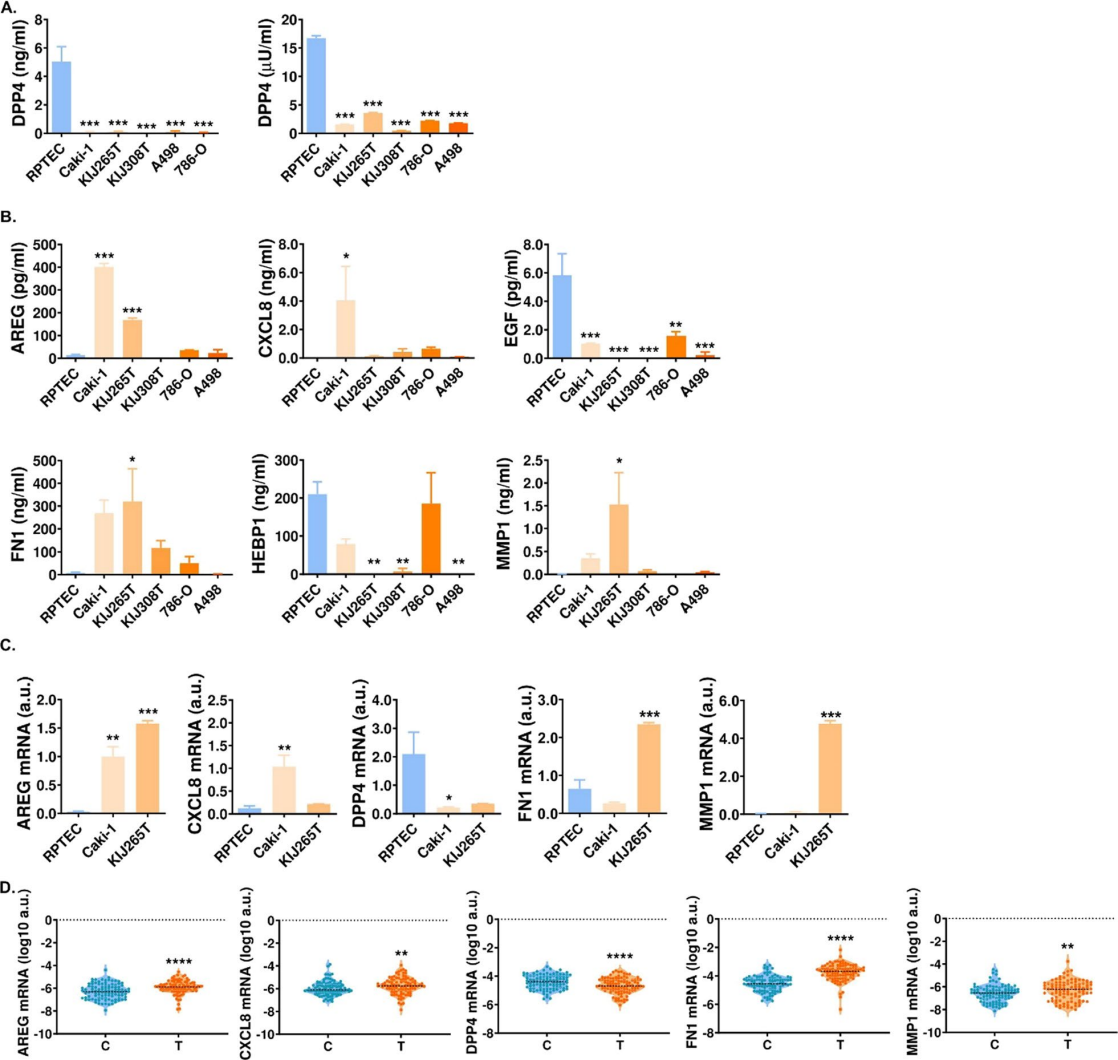
The secretomes of the advanced RCC cells share commonly altered proteins. **A** DPP4 concentration (left plot) and activity (right plot) in CM from RPTEC and RCC cell lines. **B** ELISA validation of AREG, CXCL8, EGF, FN1, HEBP1 and MMP1 in CM from RCC cell lines. **C** The expression of genes encoding cytokines is altered in RCC cell lines. The plots show the results of qPCR analysis**.** N = 3 independent biological experiments. **D** The expression of genes encoding cytokines is altered in RCC tumors. The plots show the results of qPCR analysis performed in 100 matched-paired control-tumor samples. *p < 0.05, **p < 0.01, ***p < 0.001, ****p < 0.0001. Statistical analysis was performed using one-way ANOVA with Dunnett's multiple comparisons test (A–C), paired t test or Wilcoxon matched pairs test (**D**)

Based on the results of cytokine arrays and proteomic analysis we selected AREG, CXCL8, DPP4, EGF, FN1, HEBP1 and MMP1 for ELISA validation in CM from five RCC-derived cell lines and RPTEC cells (Fig. 3). DPP4 and EGF were uniformly suppressed in CM from all analyzed RCC cell lines when compared with RPTEC (Fig. 3). In contrast, AREG, FN1 and MMP1 were selectively upregulated in CM from Caki-1 and KIJ265T cells when compared with RPTEC and the other RCC cell lines. HEBP1 and CXCL8 concentrations varied in CM depending on the RCC cell line analyzed. Altogether, these results showed that the secretomes of the advanced RCC cell lines contain specifically upregulated AREG, FN1 and MMP1.

### The expression of genes encoding cytokines is disturbed in RCC-derived cell lines and tumors

We wondered if altered secretion of cytokines could result from the changed expression of genes in RCC cell lines. qPCR confirmed altered expression of AREG, DPP4, MMP1 in Caki-1 and KIJ265T cell lines. FN1 gene expression was upregulated only in KIJ265T cells, while CXCL8 was selectively increased in Caki-1 cell lines. Furthermore, the expression of AREG, CXCL8, FN1 and MMP1 was statistically significantly increased, while the expression of DPP4 was statistically significantly decreased in RCC tumors when compared with normal kidney tissues (Fig. 3).

### AREG, FN1 and DPP4 secreted by RCC cells affect MSCs migration

**Fig. 4:**
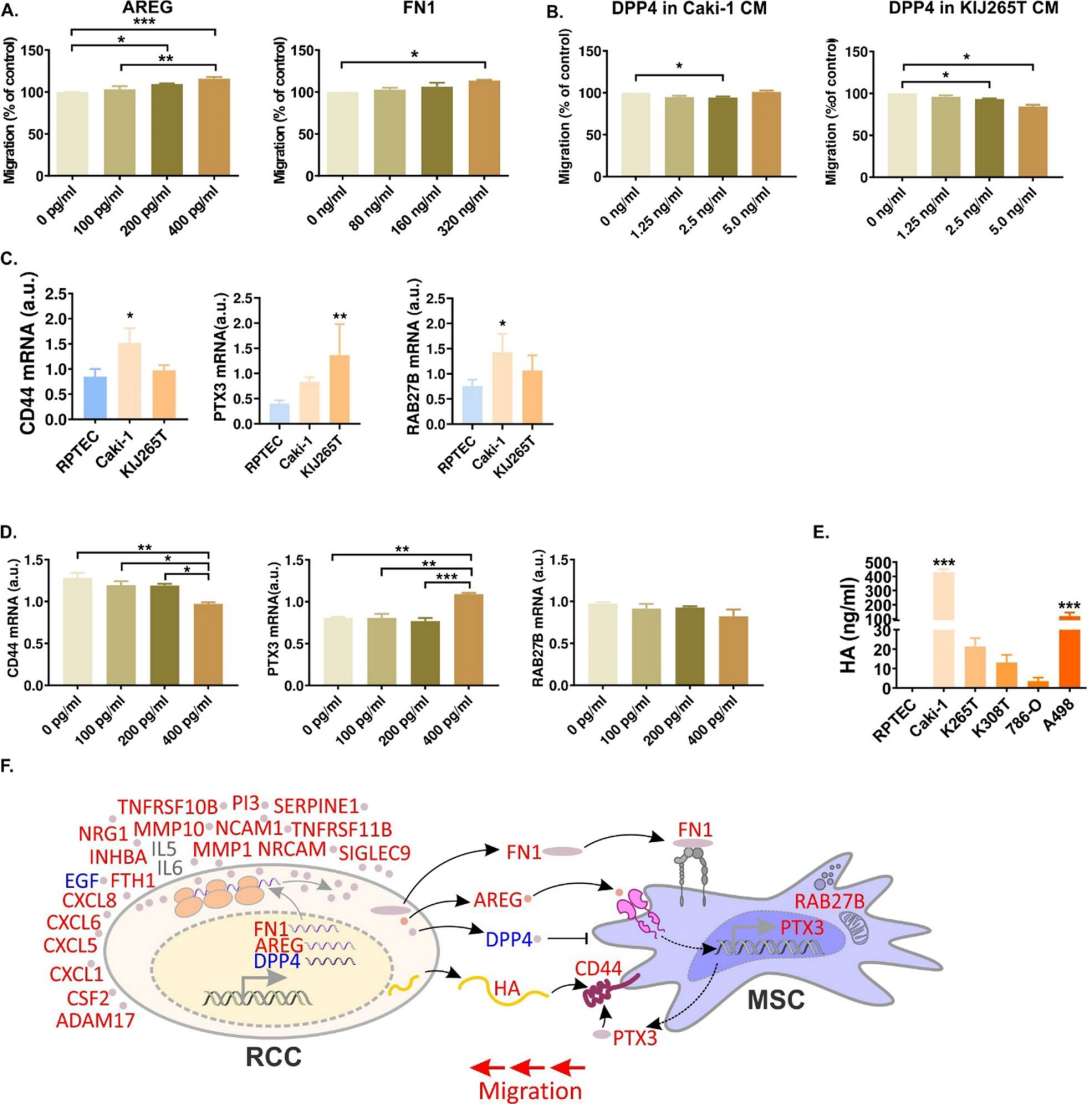
Proteins of RCC secretome stimulate MSCs migration. **A** AREG and FN1 supplementation of cell culture media induces MSC migration. **B** DPP4 supplementation of CM derived from Caki-1 (left) 17 and KIJ265T (right) inhibits MSC migration. **C** CM derived from Caki-1 and KIJ265T alter expression of MSC genes involved in migration regulation. The plots show results of three independent biological experiments performed on MSCs isolated from one patient. Statistical analysis was performed using one-way ANOVA with Dunnett's multiple comparison test **D** AREG alters expression of MSC genes involved in migration. **E** The concentration of CD44 ligand, HA, is increased in CM from RCC cell lines. The plots show results of at least three independent biological experiments. Statistical analysis was performed using One-way ANOVA with Bonferroni's multiple comparisons test (**A**,** B**) or Dunnett's multiple comparisons test (**C**–**E**). *p < 0.05, **p < 0.05, ***p < 0.001. **F** The influence of RCC secretome on MSC migration. Treatment of MSC with RCC CM stimulates their migration as well as expression of PTX3, CD44 and RAB27B. RCC cells secrete multiple cytokines/proteins that can be detected in CM. Expression of FN1 and AREG is increased, while DPP4 expression is decreased in RCC, leading to similar changes in RCC secretome. FN1 and AREG target MSC, stimulating its migration. Decreased DPP4 levels prevent its inhibitory effect on MSC motility. AREG acts on MSC, stimulating the expression of PTX3, a ligand of CD44. RCC cells secrete hyaluronic acid (HA) which binds CD44, stimulating MSC migration. Red font: expression increased; blue font: expression decreased; gray font: expression increased/decreased, depending on RCC cell line analyzed

Next, we evaluated the influence of altered RCC CM proteins on MSCs motility. Supplementation of cell culture media with AREG and FN1 stimulated MSCs migration in a dose-dependent manner (Fig. 4). DPP4 is a peptidase, therefore its enzymatic activity could affect the components of RCC secretome and thereby influence MSCs migration. Indeed, the addition of recombinant DPP4 to CM isolated from Caki-1 and KIJ265T cells suppressed MSCs migration. In contrast, neither supplementation of CM with MMP1, nor its silencing in RCC cells changed MSCs migration (Additional file 7: Fig. S2).

Altogether, these results identified AREG and FN1 as MSCs attractants, while DPP4 was confirmed as an inhibitor of MSCs migration.

### AREG is an upregulator of RCC-mediated transcriptomic MSCs reprogramming

To explore more in-depth the mechanisms of RCC secretome-mediated regulation of MSCs, we performed microarray analysis of MSCs treated with CM from Caki-1, KIJ265T and RPTEC cells (Additional file 5: Table S5). There were 55 genes with consistently altered expression in MSCs treated with CM from both RCC cell lines when compared with MSCs treated with CM from RPTEC cell lines. They included multiple genes involved in MSCs functioning, such as upregulated: CD44 (a mediator of MSCs homing to the injured kidney [15]), PTX3 (a regulator of MSCs migration, adipogenesis and MSCs-induced immunosuppression [16–18]) and RAB27B (a regulator of MSCs vesiculation and FFA metabolism [19, 20]) (Additional file 5: Table S5). qPCR confirmed statistically significantly increased expression of CD44, PTX3 and RAB27B in MSCs treated with CM from one of the analyzed RCC cell lines and a trend for increased expression in MSC treated with CM from the other RCC cell line (Fig. 4).

PTX3 was recently identified as a ligand of CD44 [21]. However, CD44 mediates MSCs homing to the injured kidney by binding hyaluronic acid (HA) [15]. We found that RCC cell lines (in particular Caki-1) secreted high amounts of HA, while its secretion by RPTEC cells was negligible (Fig. 4).

Ingenuity Pathway Analysis (IPA) predicted AREG as one of the upregulators of MSCs’ transcriptional reprogramming induced by RCC CM (Additional file 6: Table S6). Treatment of MSCs with AREG increased the expression of PTX3 and decreased the expression of CD44, while having no effect on RAB27B (Fig. 4). Altogether, these data indicated that AREG reprograms MSCs transcription to facilitate its recruitment by RCC cells.

## Discussion

In this study we show that cells of advanced renal cell carcinoma secrete cytokines and other proteins to stimulate MSCs’ motility. In particular, advanced RCC cells secrete AREG and FN1 to induce MSCs migration, while the loss of DPP4, normally secreted by healthy proximal tubules, prevents its inhibitory effect on MSCs homing. Finally, we demonstrate that RCC secretome induces reprograming of MSCs transcription, to facilitate their migration.

The novelty of our study comes from the largely underexplored mechanisms that govern MSCs’ migration. Most studies exploring the processes involved in MSC’s tumor tropism are focused on the use of MSCs for drug delivery [22]. This approach also concerns the studies on MSCs recruitment to RCC tumors. For instance, Kim et al. used MSCs co-expressing pro-apoptotic TRAIL protein and herpes simplex virus thymidine kinase (HSV-TK) with the following administration of ganciclovir (GCV) to induce apoptosis in RCC metastatic tumors inoculated in mice [8]. In this model, the MSC-TRAILHSV-TK/GCV therapy resulted in complete regression of metastatic RCC [8]. In another study, treatment of mice with MSCs producing IL-12 resulted in the reduced growth of RCC tumors and increased survival of animals [9]. Remarkably, both the abovementioned studies demonstrated that MSCs were selectively recruited to RCC tumors and/or metastatic sites in mice [8, 9]. Similar selective tropism of MSCs to RCC tumors and the sites of metastasis was shown by Hsiao et al. [7]. Using cytokine arrays they identified a PDGF-AA as a chemoattractant that induced MSCs migration toward RCC cells [7]. Lindoso et al., showed that cancer stem cells (CSCs) derived from renal cancer recruit MSCs by releasing extracellular vesicles (EVs) [23]. They also showed that the recruitment and stimulation of MSCs by CSCs-derived EVs contribute to tumor progression [23]. In conclusion, all these studies clearly indicate that MSCs are efficiently recruited to RCC tumors and can affect cancer progression; however, the specific mechanisms contributing to MSC tropism to RCC cells remain largely unknown. Regarding other types of cancer, the best-characterized cytokines that mediate MSCs homing to tumors include CXCL12, IFN-γ, IL-6, IL-8 and TNF, as well as growth factors such as TGF-β, HGF, PDGF and VEGF [22]. Our study adds now to this list AREG, FN1 and DPP4.

AREG (amphiregulin) is a ligand of EGFR which regulates MSCs’ proliferation and secretory abilities [24–27]. Cells of chronic myelogenous leukemia (CML) release AREG that targets EGFR on MSCs and alters gene expression [28]. AREG is a well-known regulator of TME. It promotes invasion of the transformed canine epithelial kidney cells [29] and was previously reported as a component of CM from Caki-1 cell line [30]. In breast cancer, it stimulates angiogenesis, regulates chemokine production and contributes to the recruitment of immune cells, thereby promoting tumor progression [31]. AREG also promotes pro-angiogenic activity of macrophages by activating VEGFA production [32]. Interestingly, MSCs stimulate pancreatic cancer (PDAC) cells to release AREG that in turn promotes PDAC invasion in an autocrine manner [33]. AREG is also secreted by TME cells, including Tregs, thereby promoting breast cancer pulmonary metastasis [34]. Fibroblasts stimulated by breast cancer cells release AREG to promote cancer cell survival [35]. AREG derived from tumor-associated dendritic cells promotes lung cancer progression [36]. All these studies indicate highly protumorous activity of amphiregulin.

We demonstrate that RCC cells from advanced tumors release FN1 to stimulate MSCs migration. This agrees with a previous study showing that MSCs interact with FN1 through α5β1 integrin receptor which results in cytoskeletal changes and facilitated migration [37]. FN1 is one of the most crucial components of the extracellular matrix (ECM), involved in the regulation of cancer progression. Secreted FN1 is an abundant plasma protein. Enhanced FN1 secretion is consistent with our previous study that showed more than fivefold increased expression of FN1 gene in RCC tumors as well as its correlation tumor grade and poor prognosis for patients [38]. Similar observations were also reported by other researchers [39, 40]. The mechanisms contributing to the dysregulated FN1 expression in RCC are complex and involve RUNX2 [41], RhoA GTPase [42], miR-1-3p [43] or hypoxia that increases deposition of FN1 fibrils in RCC cells [40]. Exogenous FN1 promotes migration and invasion of RCC cells [40]. Tissue and plasma FN1 were proposed as a RCC biomarkers [38, 39, 44, 45].

DPP4 (CD26, dipeptidyl peptidase 4) is a multifunctional glycoprotein expressed at the surface of plasma membrane. Its key function is the generation of dipeptides by cleaving peptides with terminal proline or alanine [46]. DPP4 is a powerful cytokinome regulator, capable of cleaving up to 36 chemokines and cytokines [47, 48]. It also cleaves neuropeptides and incretins [49]. Therefore, the key mechanism by which DPP4 could affect MSCs’ migration could be its influence on the homing chemokines. For instance, it is known that CXCR3 loss in MSCs attenuates their infiltration of the nephrotic kidney [50]. CXCR3 ligand, CXCL10, is cleaved by DPP4 [51]. Thus, DPP4 loss in RCC secretome may lead to the increased availability of CXCL10 for MSCs stimulating their migration toward tumor. Our cytokine arrays did not detect changes in CXCL10 in RCC secretomes. However, CXCL10 is secreted by TME cells such as dendritic cells, fibroblasts and macrophages. The specific role of DPP4 loss from RCC secretome in the context of TME requires further analysis.

What could be the causes of altered concentrations of AREG, FN1 and DPP4 in CM from RCC cells? Firstly, the expression of genes encoding all three proteins is disturbed in RCC tumors, reflecting the changed protein levels in cancer secretome (Fig. 3). Secondly, DPP4 shedding from plasma membrane is catalyzed by several metalloproteinases, including MMP1 and MMP10 [52–54], the components of RCC secretomes (Fig. 2). Thirdly, our recent study showed that expression of genes involved in protein trafficking is changed in RCC [11]. Thus, both altered gene expression and the activity of extracellular peptidases may shape RCC secretome, affecting TME, including MSCs.

Treatment of MSCs with CM from RCC cells altered the expression of genes involved in MSCs migration, including CD44 and PTX3. Earlier studies showed that CD44 interacts with hyaluronic acid to mediate MSCs’ recruitment to the injured kidney [15]. Apparently, RCC cells secrete high levels of HA, providing the ligand for MSCs’ CD44 (Fig. 4). PTX3 regulates MSCs migration, adipogenesis and MSCs-induced immunosuppression [16–18]. When expressed by CAFs, PTX3 acts as a CD44 ligand and contributes to the migration of breast cancer cells [21]. This indicates that RCC secretomes may affect MSCs to provide additional stimulation of CD44: i) by HA released by RCC cells and ii) by PTX3, expressed by MSCs in response to RCC stimuli. These effects could be in part mediated by AREG which induces PTX3 expression in MSCs. In contrast, CD44 was moderately suppressed by AREG, suggesting that other factors may contribute to the increased CD44 expression in MSCs stimulated by RCC CM. One of such factors could be PTX3 itself. PTX3 promotes HA synthesis and CD44 expression in mouse pre-osteoblasts [55]. It can be hypothesized that AREG released by RCC cells stimulates PTX3 expression in MSCs, that in turn acts in an autocrine manner (to enhance the expression of CD44 by MSCs) and in a paracrine way (to enhance HA production by RCC cells) (Fig. 4). Furthermore, PTX3 cross-links HA chains, increasing CD44 binding and downstream signaling [55]. Altogether, this suggests a possible new AREG-PTX3-CD44-HA regulatory axis contributing to the MSCs homing to RCC tumors. This interesting mechanism should be experimentally verified in future studies. Our study shows that RCC cells secrete high amounts of HA, while its secretion by normal proximal tubules was negligible (Fig. 4). This is consistent with observations performed on human patients. In the healthy kidney, HA is absent in the cortex, while is mainly produced and secreted in medullary stroma [56, 57]. This is in sharp contrast with RCC tumors in which the presence of cellular hyaluronan correlates with higher tumor grades and poor prognosis for patients. HA was also detectable in tumor stroma suggestive of its enhanced extracellular deposition [57]. RCC originates from proximal tubules which indicates significant reprogramming of HA synthesis/secretory pathway.

Our study did not aim to explore the influence of MSCs on RCC since previous studies already showed that MSCs promote renal cancer progression. MSCs stimulate proliferation and migration of RCC cells in vitro and facilitate RCC tumor formation in vivo [6, 7]. The preferable migration of MSCs toward advanced RCC suggests that MSCs may facilitate renal cancer progression. In this context, it is important to note the role of DPP4. DPP4 acts as a tumor suppressor or oncogene, depending on the study and cancer type analyzed [46]. The reports on the role of DPP4 in renal cancer provided contradicting results. Some studies showed that the expression of DPP4 mRNA and membrane-bound DPP4 protein is increased in RCC, while low DPP4 mRNA expression correlated with poor survival of RCC patients [58–60]. In contrast, other reports demonstrated the opposite results, such as decreased membrane-bound DPP4 activity in RCC and correlation of high soluble DPP4 activity (measured in tumor tissue homogenates) with tumor aggressiveness [61] and poor survival of RCC patients [62]. However, those results should be cautiously interpreted since those studies measured in fact total DPP4 plus DPP8/DPP9 activity [49]. Similar contradictory findings were reported regarding the specific role of DPP4 in RCC progression. RCC tumors express high levels of SUV39H1, a histone methyltransferase that regulates the expression of DPP4. SUV39H1 inhibition results in DPP4 upregulation and ferroptosis induction, suggestive of tumor suppressive DPP4 role [60]. On the other hand, lncRNA MCM3AP-AS1 promotes inflammation and angiogenesis in RCC tumors by stimulating DPP4 expression [63], while DPP4 silencing attenuated RCC invasion induced by ApoC1 [64]. None of those studies determined the source of sDPP4 as its activity was measured in tumor homogenates. Here, we show that sDPP4 secretion is substantially reduced in RCC when compared with normal kidney and its loss from RCC secretome contributes to MSCs migration. Taking into consideration the tumor-promoting MSCs effects in RCC, the results of our study suggest that DPP4 loss may contribute to the progression of renal cancer.

Our study provides several potential clinical implications. The identified molecules expressed/secreted by RCC cells represent potential biomarkers of tumor progression and/or targets for RCC therapy. Such therapies could involve drugs (e.g., monoclonal antibodies) inhibiting the actions of proteins inducing MSCs homing to tumor sites and leading to the attenuation of cancer progression. Furthermore, MSCs are considered in treatments of broad spectrum of conditions, including cancer [65], diabetes [66], myocardial infarction [67], bone-related dysfunctions [68] or neurodegeneration [69]. Therefore, delineation of the mechanisms that regulate MSCs’ migration is crucial for further development and improvement of therapies utilizing engineered MSCs that are currently evaluated in clinical trials [70].

There are some limitations to our study. Firstly, only one non-malignant proximal tubule cell line was used. It would be valuable to validate these results using several healthy kidney cell lines. However, even using this limited number of cell lines, the in vitro data on gene expression were accurately validated in tissue samples from human tumors. Secondly, we analyzed gene expression using the bulk RNA isolated from RCC tumors. It would be interesting to see the expressions of these genes in situ using spatial transcriptomics of tumor tissues. Thirdly, we evaluated MSCs migration only under isolated CM from RCC cell lines. Co-culture experiments involving both RCC and MSCs would provide more information on the net effects of the interactions between both types of cells. All these interesting possibilities await validation in future studies.

## Conclusions

RCC cells derived from the advanced tumors commonly share changes in cytokinome and secretome that lead to the reprograming of MSCs transcriptome and contribute to MSCs’ homing. In particular, changes in the secreted AREG, FN1, DPP4 and HA facilitate MSCs migration. In response to the AREG released by RCC cells MSCs increase the expression of PTX3 that together with HA may target CD44, contributing to MSCs motility. Considering the stimulatory MSCs effects on RCC progression as well as the utility of MSCs in drug delivery, all these molecules represent potential therapeutic targets. Finally, our study indicates that while planning MSCs’-mediated drug delivery to tumors, the composition of cancer cytokinome should be taken into consideration.

### Supplementary Information

**Additional file 1**: **Table S1**. ELISA kits used for cytokine validation.

**Additional file 2**: **Table S2**. Primers and probes used in the study.

**Additional file 3**: **Table S3**. The list of genes encoding cytokines analyzed in the study (based on https://www.raybiotech.com/c-series-human-cytokine-array-c4000-2/).

**Additional file 4**: **Table S4**. LC-MS/MS analysis of conditioned media from Caki-1 and KIJ265T cells when compared with RPTEC cell line. D: the protein was detectable in CM from the RCC-derived cell line, while it was not detectable in CM from RPTEC.

**Additional file 5**: **Table S5**. Microarray analysis of MSCs treated with CM derived from RPTEC, Caki-1 and KIJ265T cell lines.

**Additional file 6**: **Table S6**. The results of IPA predictions of the upstream regulators in MSCs treated with CM from RCC cell lines.

**Additional file 7**: **Figure S1** The representative scans of cytokine arrays performed on conditioned media retrieved from RPTEC/TERT1, Caki-1 and KIJ265T cells. Left: AAH-CYT-6 to AAH-CYT-10 represent the specific code numbers of RayBio® G-Series Human Cytokine Antibody Arrays. Chemiluminescence signals from specific cytokines are shown with red rectangles: AAH-CYT-6 : (1) EGF, (2) GCP-2/CXCL6, (3) GM-CSF, (4) IL-5, (5) IL-6; AAH-CYT-7: (1) ENA-78/CXCL5, (2) GRO alpha/CXCL1, (3) IL-8 (CXCL8), (4) OPG/TNFRSF11B; AAH-CYT-8: (1) Activin A, (2) MMP-1; AAH-CYT-9: (1) Ferritin, (2) MMP-10, (3) NCAM-1/CD56, (4) NrCAM, (5) NRG1-beta1; (6) PAI-1, (7) Siglec-9, (8) TACE, (9) TRAIL R2/TNFRSF10B, (10) Trappin-2; AAH-CYT-10: (1) CD26/DPPIV. Each antibody is spotted in duplicate vertically. **Figure S2** MMP1 secreted by RCC cells does not influence MSCs migration. A. The effects of the MMP1 supplementation of cell culture media on MSC migration. B. The effects of MMP1 silencing in RCC cells on MSC migration. Left: MMP1 concentrations in CM following silencing in Caki-1 and KIJ265T cells. Right: Migration of MSC treated with CM from Caki-1 and KIJ265T cells with silenced MMP1. The plots show results of three-to-four independent biological experiments. Statistical analysis was performed using *t* test. *p < 0.05, **p < 0.01.

## Data Availability

The data on the proteomes of conditioned media from RCC cells were published in [13] and deposited in MassIVE repository (dataset identifier PXD030085). Microarray data have been deposited in NCBI's Gene Expression Omnibus [58] and are accessible through GEO Series accession number GSE232951 (https://www.ncbi.nlm.nih.gov/geo/query/acc.cgi?acc=GSE232951).

## Abbreviations

AREG: Amphiregulin
BM-MSCs: Bone marrow MSCs
CM: Conditioned media
CML: Chronic myelogenous leukemia
DPP4: CD26, dipeptidyl peptidase 4
ECM: Extracellular matrix
FN1: Fibronectin
HA: Hyaluronic acid
mRCC: Metastatic RCC
MSCs: Mesenchymal stem/stromal cells
PDAC: Pancreatic cancer
RCC: Renal cell carcinoma
TME: Tumor microenvironment
