# Correction: Renal cancer secretome induces migration of mesenchymal stromal cells

**DOI:** 10.1186/s13287-024-03840-y

**Published:** 2024-07-23

**Authors:** Piotr Popławski, Weronika Zarychta-Wiśniewska, Anna Burdzińska, Joanna Bogusławska, Anna Adamiok-Ostrowska, Karolina Hanusek, Beata Rybicka, Alex Białas, Helena Kossowska, Roksana Iwanicka-Nowicka, Marta Koblowska, Leszek Pączek, Agnieszka Piekiełko-Witkowska

**Affiliations:** 1 Department of Biochemistry and Molecular Biology, Centre of Postgraduate Medical Education, Warsaw, Poland; 2 Present Address: Department of Immunology, Transplantology and Internal Diseases, Medical University of Warsaw, Warsaw, Poland; 3 Department of Physiological Sciences, Institute of Veterinary Medicine, Warsaw University of Life Sciences, Warsaw, Poland; 4 Laboratory of Systems Biology, Faculty of Biology, University of Warsaw, Warsaw, 02-106 Poland; 5 Laboratory of Microarray Analysis, Institute of Biochemistry and Biophysics, Polish Academy of Sciences, Warsaw, Poland; 6 Institute of Biochemistry and Biophysics, Polish Academy of Sciences, Warsaw, Poland

**Correction: Stem Cell Research & Therapy (2023) 14:200**

10.1186/s13287-023-03430-4

The original article contained a typo in co-author, Roksana Iwanicka-Nowicka’s name; the correct spelling has been restored and can also be noted in this correction article.

