# Supplementary material for: Renal cancer secretome induces migration of mesenchymal stromal cells
Source: Stem Cell Res Ther. 2023 Aug 10;14:200. doi: 10.1186/s13287-023-03430-4 (PMC10413545; doi:10.1186/s13287-023-03430-4)
Supplement: Supplementary file 1 — Additional file 1: Table S1. ELISA kits used for cytokine validation. [file 13287_2023_3430_MOESM1_ESM.docx]

**Supplementary Table S1. ELISA kits used for cytokine validation.**

| **Target protein** | **Kit** |
| --- | --- |
| EGF | DEG00 Human EGF Quantikine ELISA Kit (USA R&D Systems, Inc., Minneapolis, MN) |
| IL-8/CXCL8 | D8000C Human IL-8/CXCL8 Quantikine ELISA Kit (USA R&D Systems, Inc., Minneapolis, MN) |
| Fibronectin | DFBN10 Human Fibronectin Quantikine ELISA Kit (USA R&D Systems, Inc., Minneapolis, MN) |
| DPPIV/CD26 | DC260B Human DPPIV/CD26 Quantikine ELISA Kit (USA R&D Systems, Inc., Minneapolis, MN) |
| PEBP1 | ELH-PEBP1-1 Human PEBP1 ELISA (RayBiotech, Inc, Norcross, GA) |
| Amphiregulin | ELH-AR-1 Human Amphiregulin ELISA (RayBiotech, Inc, Norcross, GA) |
| MMP1 | EHMMP1 MMP1 Human ELISA Kit (Thermo Fisher Scientific Inc. /Life Technologies Corporation, Carlsbad, CA) |
| HEBP1 | MBS7218074 Human Heme binding protein 1 (HEBP1) ELISA Kit (MyBioSource, Inc., San Diego, CA) |
