# Supplementary material for: Renal cancer secretome induces migration of mesenchymal stromal cells
Source: Stem Cell Res Ther. 2023 Aug 10;14:200. doi: 10.1186/s13287-023-03430-4 (PMC10413545; doi:10.1186/s13287-023-03430-4)
Supplement: Supplementary file 2 — Additional file 2: Table S2. Primers and probes used in the study. [file 13287_2023_3430_MOESM2_ESM.docx]

Supplementary Table S2. Primers and probes used in the study.

| **Target** | **Primer/probe** |
| --- | --- |
| qPCR in cell lines | |
| AREG | F: GTGTCCCAGAGACCGAGTTG; R: AGGCATTTCACTCACAGGGG |
| CD44 | F: CTGCAGGTATGGGTTCATAG; R: ATATGTGTCATACTGGGAGGTG |
| CXCL8 | F: CTCCAAACCTTTCCACCCCA; R: TTCTCAGCCCTCTTCAAAAACT |
| DPP4 | F: ATGCCAGGAGGAAGGAATCT; R: TCCGGATTCAGCTCACAACT |
| FN1 | F: GACACATTCCACAAGCGTCA; R: CTCCAATTTGATAAAACGTCCC |
| MMP1 | F: ACTATTTAGAATGTAGCCCTT ; R: CATAAGCCACAAACTTGACT |
| PTX3 | F: GAGTCTTGTCACATCCGGGG; R: CAGACCTTCCCAACTGGCAT |
| RAB27B | F: GCTTGGGAAGGGGAAGGAAA; R: GATGGTCTTGGTCGGTCAGC |
| qPCR in tissue samples | |
| AREG | Hs00950669_m1 |
| CXCL8 | Hs00174103_m1 |
| DDP4 | Hs00897386_m1 |
| FN1 | Hs01549976_m1 |
| MMP1 | Hs00899658_m1 |
| RN18S1 (RNA45S5) | Hs03928985_g1 |
