# Supplementary material for: Renal cancer secretome induces migration of mesenchymal stromal cells
Source: Stem Cell Res Ther. 2023 Aug 10;14:200. doi: 10.1186/s13287-023-03430-4 (PMC10413545; doi:10.1186/s13287-023-03430-4)
Supplement: Supplementary file 7 — Additional file 7: Figure S1 The representative scans of cytokine arrays performed on conditioned media retrieved from RPTEC/TERT1, Caki-1 and KIJ265T cells. Left: AAH-CYT-6 to AAH-CYT-10 represent the specific code numbers of RayBio® G-Series Human Cytokine Antibody Arrays. Chemiluminescence signals from specific cytokines are shown with red rectangles: AAH-CYT-6 : (1) EGF, (2) GCP-2/CXCL6, (3) GM-CSF, (4) IL-5, (5) IL-6; AAH-CYT-7: (1) ENA-78/CXCL5, (2) GRO alpha/CXCL1, (3) IL-8 (CXCL8), (4) OPG/TNFRSF11B; AAH-CYT-8: (1) Activin A, (2) MMP-1; AAH-CYT-9: (1) Ferritin, (2) MMP-10, (3) NCAM-1/CD56, (4) NrCAM, (5) NRG1-beta1; (6) PAI-1, (7) Siglec-9, (8) TACE, (9) TRAIL R2/TNFRSF10B, (10) Trappin-2; AAH-CYT-10: (1) CD26/DPPIV. Each antibody is spotted in duplicate vertically. Figure S2 MMP1 secreted by RCC cells does not influence MSCs migration. A. The effects of the MMP1 supplementation of cell culture media on MSC migration. B. The effects of MMP1 silencing in RCC cells on MSC migration. Left: MMP1 concentrations in CM following silencing in Caki-1 and KIJ265T cells. Right: Migration of MSC treated with CM from Caki-1 and KIJ265T cells with silenced MMP1. The plots show results of three-to-four independent biological experiments. Statistical analysis was performed using t test. *p < 0.05, **p < 0.01. [file 13287_2023_3430_MOESM7_ESM.docx]

**Supplementary Figures**

**
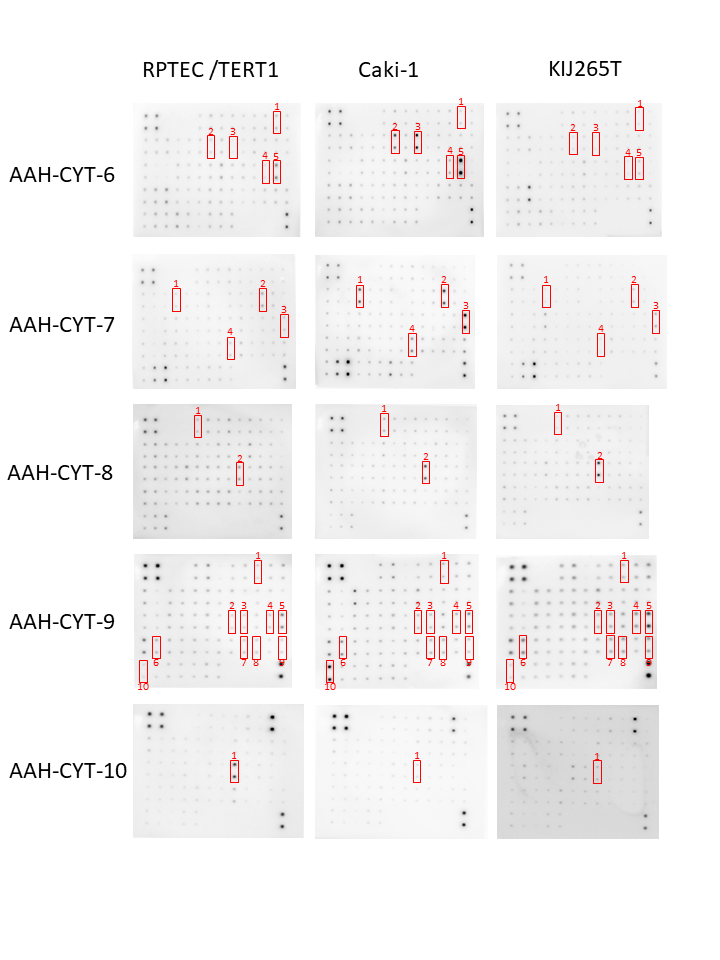
**

**Supplementary Figure S1.** The representative scans of cytokine arrays performed on conditioned media retrieved from RPTEC/TERT1, Caki-1 and KIJ265T cells. Left: AAH-CYT-6 to AAH-CYT-10 represent the specific code numbers of RayBio® G-Series Human Cytokine Antibody Arrays. Chemiluminescence signals from specific cytokines are shown with red rectangles: AAH-CYT-6 : (1) EGF, (2) GCP-2/CXCL6, (3) GM-CSF, (4) IL-5, (5) IL-6; AAH-CYT-7: (1) ENA-78/CXCL5, (2) GRO alpha/CXCL1, (3) IL-8 (CXCL8), (4) OPG/TNFRSF11B; AAH-CYT-8: (1) Activin A, (2) MMP-1; AAH-CYT-9: (1) Ferritin, (2) MMP-10, (3) NCAM-1/CD56, (4) NrCAM, (5) NRG1-beta1; (6) PAI-1, (7) Siglec-9, (8) TACE, (9) TRAIL R2/TNFRSF10B, (10) Trappin-2; AAH-CYT-10: (1) CD26/DPPIV. Each antibody is spotted in duplicate vertically.


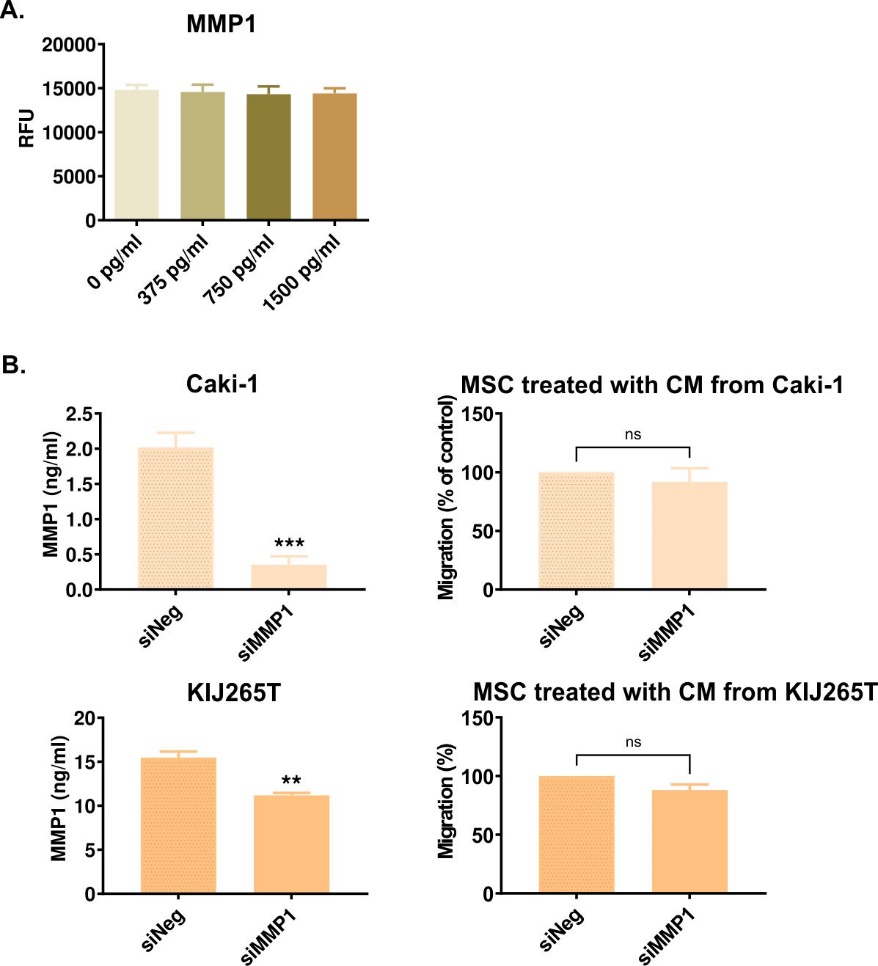


**Supplementary Figure S2.** MMP1 secreted by RCC cells does not influence MSCs migration. A. The effects of the MMP1 supplementation of cell culture media on MSC migration. B. The effects of MMP1 silencing in RCC cells on MSC migration. Left: MMP1 concentrations in CM following silencing in Caki-1 and KIJ265T cells. Right: Migration of MSC treated with CM from Caki-1 and KIJ265T cells with silenced MMP1. The plots show results of three-to-four independent biological experiments. Statistical analysis was performed using t test. * p < 0.05, ** p < 0.01.
